# Correlation and concordance of carotid Doppler ultrasound and echocardiography with invasive cardiac output measurement in critically ill patients

**DOI:** 10.1186/s40635-024-00653-4

**Published:** 2024-08-12

**Authors:** María Camila Arango-Granados, Jaime Andrés Quintero-Ramírez, Felipe Mejía-Herrera, Lina Mayerly Henao-Cardona, Valentina Muñoz-Patiño, Luis Alfonso Bustamante-Cristancho

**Affiliations:** 1 Emergency Department, Fundación Valle del Lili, Carrera 98 # 18-49, 760032 Cali, Colombia; 2 Health Sciences Faculty, Universidad Icesi, Cali, Colombia; 3 Clinical Research Center, Fundación Valle del Lili, Cali, Colombia; 4 Department of Intensive Care Unit, Clínica Imbanaco Quironsalud, Cali, Colombia

**Keywords:** Cardiac output, Carotid Doppler, Invasive hemodynamic monitoring, Ultrasound, Intensive care, Echocardiography

## Abstract

**Background:**

Critical care management heavily relies on accurate cardiac output (CO) measurement. Echocardiography has been a mainstay in non-invasive cardiac monitoring; however, its comparability to invasive methods warrants further exploration. Recent studies have suggested the potential of carotid Doppler measurements as a promising approach to estimate CO. Despite this potential, the literature presents mixed outcomes regarding its reliability and accuracy. This study aims to evaluate the correlation and concordance between carotid Doppler ultrasonography and invasive hemodynamic monitoring in estimating CO in critically ill patients. Furthermore, it assesses the concordance and correlation between echocardiography CO and the standard invasive CO measurements.

**Methods:**

This concordance study involved critically ill adults requiring invasive CO measurement. Patients with arrhythmias, severe valvulopathy, pregnancy, and poor acoustic window were excluded. Statistical analyses comprised univariate analysis, Wilcoxon signed-rank test, Spearman correlation, and intraclass correlation coefficient. Ethical approval was granted by the institution’s ethics committee.

**Results:**

A total of 49 critically ill patients were included, predominantly male (63.27%), with a median age of 57 years. Diagnoses included subarachnoid hemorrhage (53.06%) and heart failure (8.16%). Mean cardiac index was 3.36 ± 0.81 L/min/m^2^ and mean cardiac output was 5.98 ± 1.47 L/min. Spearman correlation coefficient between echocardiography and invasive CO measurements was 0.58 (*p*-value = *p* < 0.001), with an ICC of 0.59 for CO and 0.52 for cardiac index. Carotid measurements displayed no significant correlation with invasive CO.

**Conclusion:**

There is a moderate correlation and concordance between echocardiography and invasive CO measurements. There is no significant correlation between carotid variables and invasive CO, underscoring the necessity for cautious interpretation and application, particularly in patients with distinctive cerebral blood flow dynamics.

## Background

Up to one-third of critically ill patients in the intensive care unit (ICU) present with inadequate left ventricular (LV) systolic function [1]. While echocardiographic assessment remains a cornerstone for evaluating segmental and global LV motion, its accuracy depends on operator expertise [2, 3]. Despite its utility in guiding clinical decisions, complementing it with quantitative measurements is advisable [2].

The first widely used method for continuous cardiac output (CO) monitoring in the ICU was continuous thermodilution using a pulmonary artery catheter [4]. However, the lack of beneficial effects on mortality and patient outcomes, coupled with safety concerns, has led to a decline in its use [5]. With the interest in minimally invasive hemodynamic monitoring, new methods such as pulse contour analysis, aortic flow, bioimpedance, and bioreactance have emerged, each with varying accuracy [4]. Arterial contour devices estimate CO from the arterial pulse using a transfer function that presumes specific arterial-vascular compliance. These devices rely on assumptions about arterial dynamics, which can lead to potential inaccuracies, especially in cases of  arrhythmias, aortic regurgitation, abnormal systemic vascular resistance, and the use of an intra-aortic balloon pump [4, 6]. Thoracic bioimpedance, though non-invasive, is often inaccurate in acute-care settings due to electrical interference. In contrast, bioreactance, which uses frequency- and phase-modulation, significantly reduces the impact of electrical fields on CO estimates and has shown robust performance across various conditions [4, 7].

Echocardiography, traditionally used for diagnostic purposes, has recently gained prominence as a real-time diagnostic tool in critical care, allowing rapid assessment and reassessment of patients post-intervention or during significant clinical changes. Acknowledged by the European Society of Intensive Care, echocardiography stands as the preferred modality for guiding diagnosis and treatment of patients in shock [8]. The conventional approach for CO measurement via echocardiography entails assessing the left ventricular outflow tract (LVOT). By determining LVOT diameter and Velocity Time Integral (VTI) with pulsed Doppler in the LVOT, CO can be computed using the formula CO = LVOT VTI x LVOT cross-sectional area x heart rate [2]. LVOT VTI serves as a surrogate for systolic volume, with values below 18 cm linked to adverse outcomes such as heart failure, hospitalization, and mortality [9].

Despite echocardiography’s recognized reproducibility and reliability in CO measurement since the 1990s, challenges persist [10]. These include the need for specialized training, difficulty in patients with suboptimal acoustic windows, constraints in maintaining consistent angle between the ultrasound beam and the LVOT, and time-consuming measurements procedures. Moreover, while LVOT CO measurements have shown reproducibility and reliability [10], discrepancies with traditional invasive CO measurement techniques are common. Observation suggests echocardiography tends to underestimate true CO, possibly attributable to alignment issues encountered during measurement. Moreover, while many studies have examined the concordance between echocardiography and invasive CO measurements, few have delved into the absolute agreement between the two modalities.

To address these challenges, alternative techniques for CO measurement via ultrasound (US) have gained attention. Among these, carotid flow measurement stands out as the most investigated. However, studies assessing its reproducibility and validity have yielded conflicting findings [11–20]. Therefore, the primary objective of this study is to assess the correlation and concordance between carotid Doppler measurements and invasive hemodynamic monitoring for CO estimation in critically ill patients. Additionally, the study aims to evaluate the correlation and concordance between standard echocardiography CO measurement techniques and invasive CO measurement methods.

## Methods

### Study design

This prospective study employed a correlation and concordance analysis approach, aiming to assess the reliability of non-invasive methods for measuring CO in critically ill patients. Conducted at Fundación Valle del Lili in Cali, Colombia, a tertiary care center renowned for its expertise in critical care and advanced diagnostic capabilities.

### Population and sample

A non-probabilistic quota sampling method was employed to determine the sample size, enrolling 49 critically ill patients aged 18 years and above, who required invasive CO measurement during their ICU stay. Invasive monitoring systems such as Swan-Ganz catheters, PiCCO and EV-1000 systems were utilized for CO measurement. Patients with active arrhythmias, severe mitral or aortic valve disease, pregnancy, or inadequate acoustic window for carotid Doppler assessment were excluded from the study.

### Operational aspects

Eligible patients underwent evaluation in the ICU, where an external observer recorded general data and CO measurements obtained via invasive methods. Following this, an independent observer, blinded by the invasive CO results, conducted the US assessments. These measurements were carried out by three US experienced physicians with varying levels of expertise including an emergency medicine resident, a junior emergency physician, and an emergency physician specializing in intensive care and radiology. Each patient was examined by one of the three physicians.

The measurements were performed at predetermined times during the patients’ ICU stay, after invasive CO measurements were conducted. All patients underwent both echocardiography and carotid ultrasound measurements. Each ultrasound measurement was taken as an average of 3 to 5 consecutive heartbeats.

Utilizing a high-frequency linear transducer, the common carotid artery (CCA) was identified bilaterally in both transverse and longitudinal planes using grayscale ultrasound. Subsequently, spectral waveforms of blood flow velocity within the proximal CCA were captured using pulse wave Doppler, with precise placement of the sample volume approximately 1–2 cm below the carotid bulb. Doppler angle adjustment was optimized to approach 0 °. The obtained variables included carotid artery diameter, systo-diastolic time average peak (TAP) velocities, systolic TAP velocities, systo-diastolic flow, and systolic flow.

For the estimation of cardiac output through echocardiography, a phased-array transducer was employed. The parasternal long-axis window was utilized to measure the LVOT diameter, and the apical five-chamber view was employed to capture the VTI of blood flow through the LVOT using pulse wave Doppler. LVOT VTI measurements were conducted both with and without angle correction.

### Statistical analysis

An exploratory analysis of the data was conducted, along with an assessment of data quality through a randomized probabilistic sampling of 10% of the records to ensure consistency and accuracy. Univariate analysis was employed to assess the distribution of numerical variables using the Shapiro–Wilk test, with results summarized as mean ± standard deviation (SD) or median ± interquartile ranges (IQR) as appropriate. Categorical variables were presented as absolute numbers (n) and relative frequencies (%).

The comparison between right and left carotid Doppler ultrasound measurements was conducted using the Wilcoxon signed-rank test. Correlation analysis between CO, cardiac index (CI), and systo-diastolic flow obtained via echocardiography and carotid Doppler ultrasound was performed using the Spearman coefficient, with all invasive CO measurement as the reference standard for comparison. Correlation coefficients falling within the ranges of 0.00–0.09 were considered negligible, 0.10–0.39 weak, 0.40–0.69 moderate, 0.70–0.89 strong, and 0.90–1.00 very strong [21].

Concordance analysis between CO and CI was assessed using the intraclass correlation coefficient (ICC), with all invasive CO measurement serving as the reference standard. ICC values below 0.5 indicated poor reliability, those between 0.5 and 0.75 indicated moderate reliability, those between 0.75 and 0.9 indicated good reliability, and those exceeding 0.90 indicate excellent reliability [22].

Statistical significance was defined as a *p*-value < 0.05. All statistical analyses were performed using Stata version 14 (StataCorp LP, College Station, TX).

### Ethical considerations

Approval for this study was obtained from the ethics committee of Fundación Valle del Lili, adhering to international recommendations regarding research involving human subjects, including compliance with the Nuremberg Code, the Helsinki Declaration, and the guidelines of the CIOMS. Patient confidentiality was rigorously maintained and individuals were afforded the right to decline participation without any repercussions on their medical treatment.

## Results

The study included 49 critically ill patients with a median age of 57 years (range: 37–68). Males constituted 63.27% of the cohort, while females represented 36.73%. The main diagnoses varied, with subarachnoid hemorrhage being the most prevalent (53.06%), followed by decompensated heart failure (8.16%), and severe multiorgan dysfunction syndrome/severe COVID19-associated acute respiratory distress syndrome (8.16%). Cardiac output monitoring in these patients was primarily indicated due to vasospasm or high risks of vasospasm (48.9%), and various forms of shock, including distributive (16.3%), cardiogenic (8.2%), hypovolemic (4.1%), vasoplegic (2%), mixed (2%) and unclassified shock (12.2%), as well as stress cardiomyopathy (2%). Notably, 30 patients (61.2%) were on vasopressors and/or inotropes. The primary invasive monitoring system utilized was PiCCO2 (63.3%), followed by EV-1000 (22.4%) and Swan-Ganz (14.3%). Hemodynamic assessments revealed a mean CI of 3.36 ± 0.81 L/min/m^2^ and a mean cardiac output of 5.98 ± 1.47 L/min. Additional demographic and clinical characteristics are summarized in Table 1.

**Table 1:** Characteristics of the study population (*n* = 49)

Age**	57 (37–68)
Sex, n (%)	
Female	18 (36.73)
Male	31 (63.27)
Main diagnoses, n (%)	
Subarachnoid hemorrhage	26 (53.06)
Decompensated heart failure Stevenson B	4 (8.16)
Multiorgan dysfunction syndrome/severe COVID19-associated acute respiratory distress syndrome	4 (8.16)
Severe diabetic ketoacidosis / hyperosmolar state	3 (6.12)
Cardiac revascularization	3 (6.12)
Acute Respiratory Failure/Heart Failure	2 (4.08)
Gastrointestinal tract hemorrhage	1 (2.04)
Acute hydrocephalus due to DVP dysfunction	1 (2.04)
Acute myocardial infarction	1 (2.04)
Polytrauma due to traffic accident	1 (2.04)
Postoperative pancreatoduodenectomy	1 (2.04)
Postoperative aortic valve replacement	1 (2.04)
Thoracic aortic aneurysm rupture STANFORD A	1 (2.04)
Vasoactive agents	
Vasopressors	23 (46.9)
Inotropes	19 (38.7)
Vasodilators	12 (24.5)
None	4 (8.2)
No information	3 (6.1)
Type of invasive system	
PiCCO2	31 (63.3)
EV-1000	11 (22.4)
Swan-Ganz	7 (14.3)
Weight (kilos) **	75 (65–84)
Height (Centimeters)**	162 (155–168)
Cardiac index (L/min/m^2^)^$^*	3.36 ± 0.81
Cardiac output (L/min)^%^*	5.98 ± 1.47

^*^Mean ± SD^**^Median (IQR) $ L/min/m^2^: Liters per minute per square meter of body surface area. % *L/min* Liters per minute

**Fig. 1:**
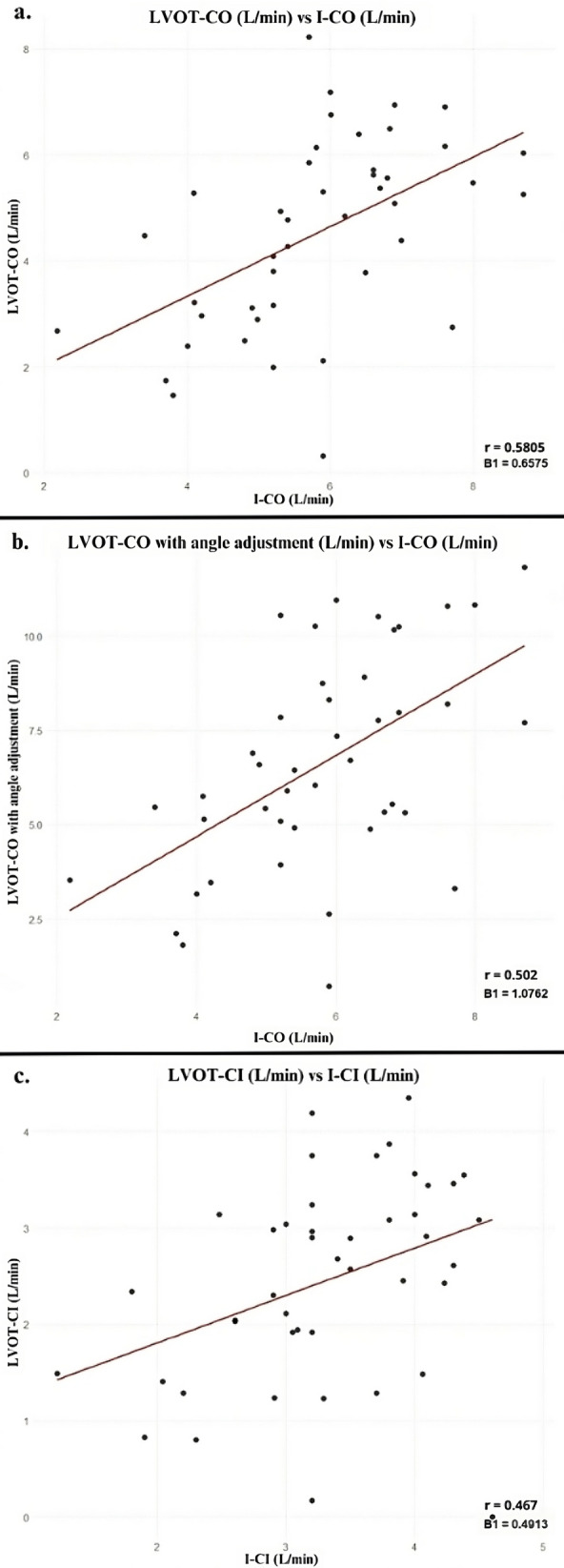
Correlations between LVOT and invasive measurements. Scatterplots showing the correlations between LVOT and invasive measurements. Parameters include CO (**a**), CO with angle adjustment (**b**), and CI (**c**). LVOT-CO Left ventricular outflow tract cardiac output, I-CO Invasive cardiac output, LVOT-CI Left ventricular outflow tract cardiac Index, I-CI Invasive cardiac Index

**Fig. 2:**
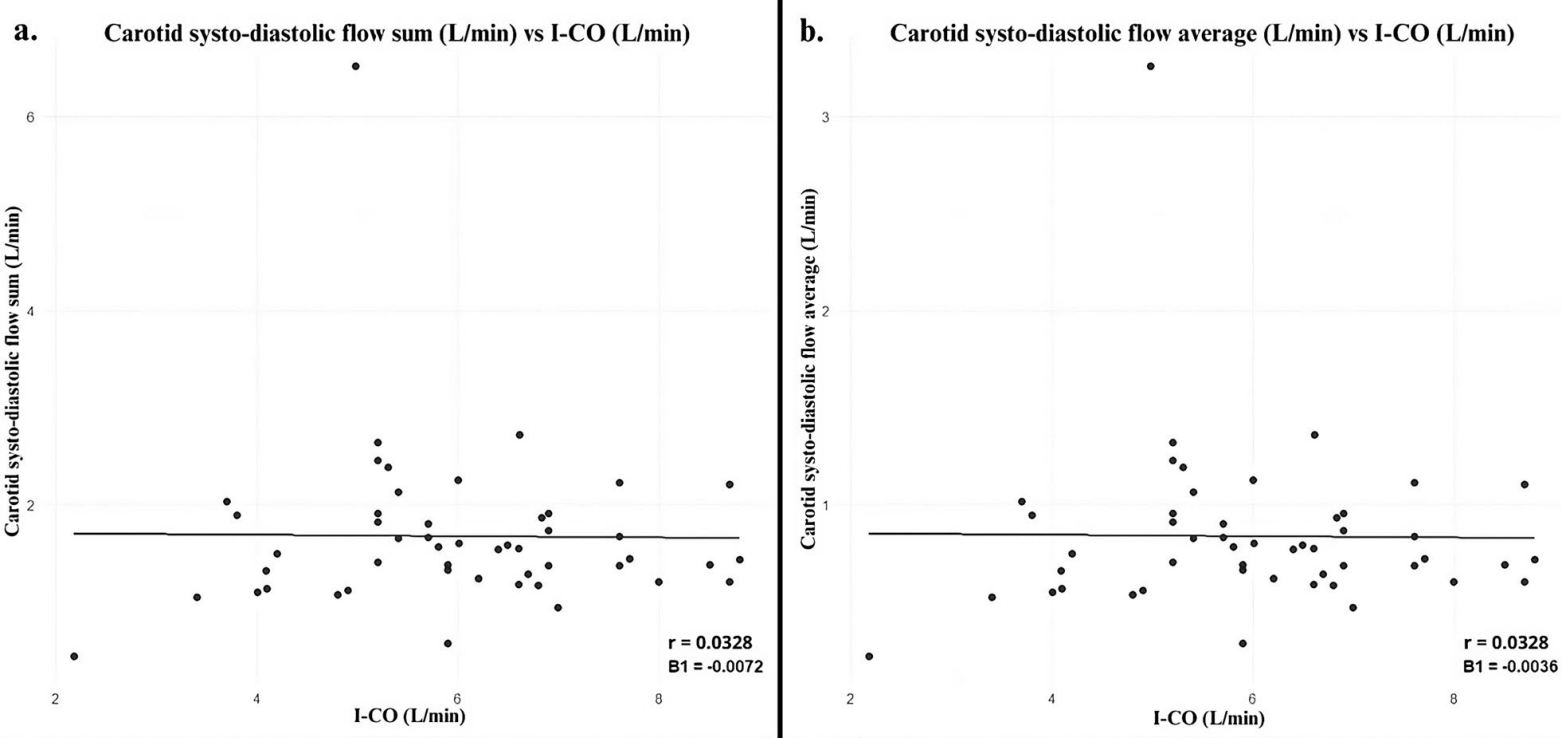
Correlations between carotid ultrasound and invasive measurements**.** Scatterplots showing the correlations between carotid ultrasound and invasive measurements. Parameters include sum of carotid systo-diastolic flows (**a**), and average of carotid systo-diastolic flows (**b**). I-CO Invasive cardiac output

The Spearman correlation coefficient indicate a moderate positive correlation between LVOT and invasive CO measurement (*r* = 0.580, *p*-value < 0.001) and between LVOT and invasive CI measurement (*r* = 0.467, p-value = 0.0014) (Table 2). After performing angle adjustment of the LVOT VTI measurement, the correlation with invasive CO measurement did not improve (*r* = 0.502, *p* = 0.0006). Concordance analysis showed moderate concordance between LVOT and invasive CO measurement (ICC = 0.59), and between LVOT and invasive CI measurement (ICC = 0.524) (Fig. 1). The sum and average of carotid systo-diastolic flows showed negligible correlation with invasive CO (*r* = 0.033, p-value > 0.05) suggesting limited usefulness of these measurements to predict invasive CO in this patient sample (Fig. 2).

**Table 2:** Correlations and concordances between ultrasonographic and invasive measurements

	Spearman correlation	*p*-value	Concordance (ICC)
LVOT-CO measurement (L/min) vs invasive CO measurement (L/min)	0.5805	*p* < 0.001	0.5900594
LVOT-CO measurement with angle adjustment (L/min) vs invasive CO measurement (L/min)	0.502	0.0006	0.529488
LVOT-CI measurement (L/min) vs invasive CI measurement (L/min)	0.467	0.0014	0.5237697
Sum of carotid systo-diastolic flows (L/min) vs invasive cardiac output (L/min)	0.0328	0.8228	
Average systo-diastolic carotid flows (L/min) vs invasive cardiac output (L/min)	0.0328	0.8228	

*LVOT* Left ventricular outflow tract, *CO* Cardiac output, *CI* Cardiac index, *ICC* Intraclass correlation coefficient

Given the high proportion of patients with subarachnoid hemorrhage, a subanalysis was performed to compare the correlations and concordances between US and invasive measurement in neurocritical patients (*n* = 27) and patients with other pathologies (*n* = 22). The results for both groups were similar to the results for the entire cohort (Table 3).

**Table 3:** Correlations and concordances between ultrasonographic and invasive measurements of patients with neurocritical and non-neurocritical pathologies

Neurocritical pathologies (*n* = 27)			
Parameter	Spearman correlation	*p*-value	Concordance (ICC)
LVOT-CO measurement (L/min) vs invasive CO measurement (L/min)	0.5099	0.0257	0.6897822
LVOT-CO measurement with angle adjustment (L/min) vs invasive CO measurement (L/min)	0.466	0.0443	0.6317556
LVOT-CI measurement (L/min) vs invasive CI measurement (L/min)	0.5248	0.0211	0.7551931
Sum of carotid systo-diastolic flows (L/min) vs invasive cardiac output (L/min)	0.0994	0.6598	
Average systo-diastolic carotid flows (L/min) vs invasive cardiac output (L/min)	0.0994	0.6598	
Non—neurocritical pathologies (*n* = 22)			
LVOT-CO measurement (L/min) vs invasive CO measurement (L/min)	0.5661	0.0039	0.5436345
LVOT-CO measurement with angle adjustment (L/min) vs invasive CO measurement (L/min)	0.4714	0.02	0.3902596
LVOT-CI measurement (L/min) vs invasive CI measurement (L/min)	0.4362	0.0293	0.2545285
Sum of carotid systo-diastolic flows (L/min) vs invasive cardiac output (L/min)	0.0309	0.8784	
Average systo-diastolic carotid flows (L/min) vs invasive cardiac output (L/min)	0.0309	0.8784	

*LVOT* Left ventricular outflow tract, *CO* Cardiac output, *CI* Cardiac index, *ICC* Intraclass correlation coefficient

A correlation analysis was conducted between carotid ultrasound variables (including carotid diameter, TAP, flows, and heart rate) and invasive CO. However, none of these correlations reached statistical significance.

## Discussion

Our correlation and concordance analysis between LVOT CO measurements and those derived from invasive techniques challenges conventional expectations from prior literature. The moderate correlation and concordance observed raise doubts about the efficacy of echocardiography in predicting CO in critically ill patients, contrasting with previous studies lauding its utility and precision. Previous research, including studies by Coats AJ (1990) and Cecconi et al. (2014), consistently emphasized echocardiography’s accuracy and reliability for hemodynamic monitoring [8, 10]. However, our findings suggest suboptimal correlation and concordance with invasive measures, potentially due to technical limitations, operator proficiency disparities, or patient heterogeneity.

Angle adjustment of LVOT Doppler measurements did not improve the correlations, contrary to expectations. This adjustment was initially hypothesized to rectify potential inaccuracies in measurements, as it typically enhances the certainty of Doppler measurements. This underlines the importance of context considerations in interpreting cardiac US results and suggests the potential need for standardized protocols or specific training to enhance accuracy.

On the other hand, the lack of significant correlation between carotid measurements and invasive CO underscores a notable limitation in the predictive capacity of carotid measurements for CO assessment. While carotid flow measurement has gained attention as a promising technique, with prior studies by Peng QY et al. (2017) and Ma IWY et al. (2017) reporting positive correlations [17, 18], our study did not reproduce these findings. This discrepancy may stem from the heterogeneity of the study population, differences in measurement methodologies, or the influence of uncontrolled confounding variables.

The anatomical characteristics of carotid arteries as extrathoracic vessels introduce unique considerations regarding their utility in reflecting CO. Unlike intrathoracic vessels, whose flow dynamics are directly influenced by cardiac function and central hemodynamic conditions, carotid artery flow is also influenced by extracardiac factors such as peripheral vascular resistance and cerebral blood flow dynamics [23]. These external factors may alter the CO-carotid flow relationship, potentially attenuating any direct correlation between the two.

The notable representation of neurocritical patients in our study introduces a noteworthy variable for assessing the reliability of carotid measurements in predicting CO. Neurocritical patients, particularly those afflicted with conditions such as subarachnoid hemorrhage, head trauma, or cerebrovascular disease, exhibit distinct cerebral blood flow dynamics regulated by cerebral autoregulation and intracranial pressure (ICP) fluctuations, which can profoundly influence carotid flow [24, 25]. Cerebral autoregulation, responsible for maintaining constant cerebral blood flow despite changes in systemic blood pressure, may be compromised in individuals with acute brain injury [24]. Consequently, any fluctuations in blood pressure could exert a more pronounced and direct effect on cerebral blood flow, thereby impacting carotid Doppler measurements.

Prior research has shown that increased ICP or disruptions in cerebral autoregulation can induce substantial changes in blood flow, which may not accurately reflect the overall hemodynamic status of the patient but rather the localized cerebral flow dynamics [26, 27]. Moreover, the phenomenon of vasospasm, prevalent among patients with subarachnoid hemorrhage, can reduce cerebral blood flow in downstream region [25], which can directly impact carotid flow measurements and complicate the accurate estimation of CO.

These pathophysiological mechanisms in neurocritical patients suggest that carotid flow measurements may not accurately reflect CO, as they can be influenced by cerebral dynamics rather than solely cardiac function. This underscores the importance of exercising caution when interpreting carotid flow measurements in this patient subgroup and considering adjustments or predictive models that account for the particularities of cerebral pathophysiology when estimating CO. Despite conducting a subanalysis of patients with non-neurological critical conditions to mitigate this bias, the findings remained consistent with those of the overall cohort. Future investigations should aim to develop tailored approaches to hemodynamic monitoring in neurocritical patients that integrate assessments of both global cardiac function and localized cerebral blood flow alterations.

This study aimed to employ logistic regression, incorporating carotid variables to predict CO. However, none of the correlation tests between these variables (diameter, TAP and flow) and CO were significant. The absence of significant correlations between these carotid variables and invasive CO strongly argues against implementing logistic regression models for CO prediction. Logistic regression relies on significant relationships between independent variables (predictors) and the dependent variable (outcome). Significance in initial correlations not only suggests potential linear relationships but also provides a theoretical basis for exploring such relationships in more complex predictive models. Without such significance, any regression model would lack a robust statistical foundation, risking erroneous interpretations or overestimation of predictive capabilities of the variables [28]. Furthermore, continuing regression without significant correlations heightens the risk of Type I errors (false positives). Opting not to proceed with logistic regression demonstrates methodological rigor and a cautious interpretation of preliminary data.

## Strengths and limitations

The present study possesses several strengths. Firstly, a meticulous methodological approach to data collection and analysis was adopted, including the use of blinded observers to gold standard measurements to mitigate bias. Secondly, the inclusion of operators with varying levels of expertise not only enhances the relevance of findings to real-world clinical scenarios but also underlines the significance of operator proficiency as a variable in assessing the reliability of ultrasound techniques.

The identified weaknesses of the study are delineated as follows: firstly, the absence of significant correlations between carotid measurements and invasive cardiac output restrained the study’s capacity to construct robust predictive models, thereby undermining the study’s primary objective. Secondly, despite the inclusion of patients with diverse diagnoses, the predominance of neurocritical cases may have directly influenced the predictive efficacy of carotid measurements. Thirdly, the accuracy of echocardiographic and carotid measurements is markedly contingent on image quality and operator proficiency, variables that can exhibit considerable variability and thereby impact measurement precision.

## Conclusions

Contrary to anticipated outcomes based on prior literature, our study revealed moderate correlation and concordance between echocardiography CO measurements and those acquired via invasive techniques. Furthermore, the lack of significant correlations between carotid US variables and invasive CO underscores the complexity of interpreting carotid measurements in critically ill patients, particularly those with neurocritical conditions affecting cerebral blood flow dynamics. It is imperative that future investigations focus on refining clinically relevant and statistically validated methodologies, and predictive models to enhance hemodynamic monitoring and optimize management strategies for critically ill patients. This approach will ensure that clinical decisions are firmly grounded in evidence-based practices, thereby optimizing patient outcomes in critical care settings.

**Take-home message** Contrary to previous beliefs, our study found only moderate correlation between echocardiography and invasive techniques for cardiac output measurement in critically ill patients. Carotid measurements showed no significant correlation with invasive cardiac output.

## Data Availability

The datasets used and analyzed during the current study are available from the corresponding author on reasonable request.

## Abbreviations

ICU: Intensive care unit
LV: Left ventricular
CO: Cardiac output
US: Ultrasound
LVOT: Left ventricular outflow tract
VTI: Velocity time integral
CCA: Common carotid artery
TAP: Time average peak
SD: Standard deviation
IQR: Interquartile ranges
CI: Cardiac Index
ICC: Intraclass correlation coefficient
LVOT-CO: Left ventricular outflow tract cardiac output
I-CO: Invasive cardiac output
LVOT-CI: Left ventricular outflow tract cardiac Index
I-CI: Invasive cardiac index
ICP: Intracranial pressure
